# Application of remote sensing and geospatial technologies in predicting vector-borne disease outbreaks

**DOI:** 10.1098/rsos.250536

**Published:** 2025-10-15

**Authors:** Ebrahim Abbasi

**Affiliations:** ^1^ Biology and Control of Disease Vectors, Shiraz University of Medical Sciences, Shiraz, Fars, Iran

**Keywords:** vector-borne diseases, remote sensing, geospatial technologies, predictive modelling, geographical information systems (GIS), early warning systems, environmental health

## Abstract

Vector-borne diseases (VBDs) pose significant global health threats, particularly in tropical and subtropical regions. Remote sensing (RS) and geospatial technologies offer valuable tools for monitoring environmental changes and predicting disease transmission patterns, thereby supporting proactive public health interventions. This study reviews the application of RS and geospatial methods in the prediction, monitoring and control of VBDs. A systematic approach was employed to analyse existing literature, focusing on RS platforms such as Landsat, MODIS and Sentinel-2, alongside geographical information systems and machine learning models used for predictive modelling. The review reveals that these technologies play a crucial role in identifying environmental drivers of disease dynamics, including temperature, precipitation and land-use changes. However, challenges remain in terms of data resolution, model generalizability and the integration of socio-economic factors into predictive frameworks. The integration of early warning systems and participatory surveillance is highlighted as a promising avenue for improving disease forecasting. The study emphasizes the need for enhanced data accessibility, cross-sector collaboration and the inclusion of socio-economic variables in future research to improve the scalability and accuracy of disease prediction models.

## 1. Introduction

Vector-borne diseases (VBDs) continue to represent a major global health challenge, affecting millions of individuals annually and posing significant threats to public health systems, particularly in tropical and subtropical regions. These diseases are transmitted by vectors such as mosquitoes, ticks and flies, with pathogens like viruses, bacteria and protozoa causing widespread morbidity and mortality. The emergence and spread of VBDs are influenced by a range of complex environmental, climatic and socio-economic factors. Understanding and predicting the spatial and temporal dynamics of these diseases is critical for implementing timely and effective control measures, particularly in the context of rapid urbanization, climate change and increasing global mobility [1–6].

Over the past few decades, advances in geospatial technologies and remote sensing (RS) have revolutionized the way researchers monitor and analyse the environmental and ecological factors contributing to VBD transmission. RS data, when integrated with geographical information systems (GIS), provide powerful tools for the analysis of vector habitats, the identification of risk zones and the prediction of disease outbreaks. These technologies offer a unique opportunity to gather high-resolution, large-scale data on environmental variables such as temperature, humidity, precipitation, land use, vegetation cover and topography, all of which are crucial in understanding vector distribution and behaviour [7–11].

One of the key advantages of using RS and geospatial technologies in the study of VBDs lies in their ability to provide real-time, continuous and global coverage. Unlike traditional ground-based surveillance methods, which are often time-consuming, resource-intensive and geographically limited, RS allows for the monitoring of vast and inaccessible areas. This capacity is especially important in regions where disease outbreaks are difficult to predict owing to poor infrastructure or limited surveillance systems. The integration of Earth observation (EO) data with epidemiological models enhances the accuracy of disease forecasting and risk assessments, leading to improved early warning systems (EWS) and proactive health interventions [12–14].

Recent studies have highlighted the potential of geospatial data in identifying environmental risk factors that influence the presence and spread of vector populations. For example, temperature and rainfall patterns directly affect the breeding and survival rates of many vector species, with variations in these factors often leading to changes in the geographical distribution of disease vectors. Additionally, land use changes, such as urbanization, deforestation and agricultural expansion, can alter the ecological balance, creating new breeding habitats for vectors. By using geospatial data, researchers can track these environmental changes over time, thereby providing early indicators of potential outbreaks [15–17].

In recent years, the application of geospatial technologies has significantly contributed to the development of predictive models that can estimate the risk of VBD outbreaks in specific regions. These models incorporate multiple variables, such as climatic conditions, population density and historical disease data, to predict the likelihood of disease transmission in a given area. Furthermore, the combination of RS data with machine learning (ML) and artificial intelligence (AI) techniques holds immense promise for enhancing predictive accuracy. These advanced analytical approaches can identify complex patterns and trends that may not be immediately apparent through traditional statistical methods [12,18,19].

Despite the promising advancements in geospatial and RS technologies, several challenges remain in their application to VBD research. One of the primary challenges is the need for high-quality, high-resolution data that can accurately represent local environmental conditions. Although satellite imagery and airborne sensors provide valuable data, issues such as cloud cover, spatial resolution and temporal frequency can limit their use in certain regions. Additionally, the integration of EO data with epidemiological data requires robust modelling techniques that can account for the complex interactions between environmental factors, vector behaviour and disease transmission dynamics [16,20,21].

Furthermore, there is a need for interdisciplinary collaboration between entomologists, epidemiologists, climatologists and geospatial scientists to fully realize the potential of these technologies in combating VBDs. Effective data sharing, standardization of methodologies and the development of user-friendly decision support systems are also essential for translating research findings into actionable health policies and interventions [22–28].

This review aims to explore the current applications and future potential of RS and geospatial technologies in predicting and managing VBD outbreaks. By examining the latest research and case studies, this study seeks to highlight the critical role of EO data in enhancing our understanding of the environmental factors that drive the spread of VBDs. Moreover, this review will identify key challenges and opportunities for advancing the integration of geospatial technologies into public health strategies, particularly in the context of changing environmental and climatic conditions. In doing so, it will contribute to the growing body of knowledge on the role of RS in improving disease surveillance, EWS and control measures for VBDs [29–31].

## 2. Material and methods

This review adopts a systematic and integrative approach to assess the current applications and future potential of RS and geospatial technologies in predicting and managing VBD outbreaks. The methodology is designed to ensure a rigorous synthesis of the existing body of literature, focusing on interdisciplinary studies that bridge the fields of medical entomology, RS and geospatial analysis. The following subsections outline the key steps and criteria employed in the review process, ensuring transparency, reproducibility and scientific rigour.

### 2.1. Literature search strategy

To gather relevant literature, a systematic search was conducted across multiple scientific databases, including PubMed, Web of Science, Scopus and IEEE Xplore. These databases were selected for their comprehensive coverage of biomedical, environmental and technological research. The search strategy was tailored to include keywords and Boolean operators to capture the intersection of topics related to VBDs, RS and geospatial technologies. Examples of search terms included, ‘Vector-borne diseases AND remote sensing’, ‘Geospatial analysis AND disease outbreaks’, ‘Earth observation data AND vector ecology’, ‘GIS AND arboviral disease prediction’. The search was restricted to peer-reviewed articles published between 2000 and 2025 to ensure the inclusion of contemporary advancements in the field. Articles published in English were prioritized to maintain consistency in language and interpretation. Additionally, reference lists of key articles were screened to identify relevant studies that may not have been captured in the initial search [16,32,33].

### 2.2. Inclusion and exclusion criteria

To ensure the relevance and quality of the studies included in the review, predefined inclusion and exclusion criteria were applied. Studies were considered eligible if they met the following criteria, focused on the application of RS or geospatial technologies in the context of VBDs, addressed environmental, climatic or ecological factors influencing vector distribution or disease transmission, provided quantitative or qualitative insights into predictive modelling, risk assessment or EWS for VBDs, published in peer-reviewed journals and available in full-text format. Exclusion criteria included; studies that solely focused on laboratory-based vector research without a geospatial or environmental component, articles lacking sufficient methodological detail or transparency, reviews or meta-analyses that did not contribute novel perspectives to the topic. All titles and abstracts were independently screened by two reviewers to ensure consistency and minimize bias. Discrepancies were resolved through discussion or consultation with a third reviewer [34–39].

### 2.3. Data extraction and synthesis

A structured data extraction template was developed to capture relevant information from the selected studies. Extracted data included study objectives and research questions, geographical location and disease focus (e.g. malaria, dengue, Zika virus), RS platforms and datasets used (e.g. MODIS, Landsat, Sentinel), analytical techniques employed, such as GIS-based mapping, ML models or spatial statistics, key findings and implications for vector surveillance and control. To synthesize the extracted data, studies were grouped into thematic categories based on their primary focus, such as environmental monitoring, predictive modelling or integration of RS with public health strategies. Within each category, findings were analysed to identify recurring patterns, gaps and emerging trends [40–45].

### 2.4. Quality assessment

To ensure the reliability and validity of the included studies, a quality assessment framework was applied. The framework was adapted from established guidelines for systematic reviews and included the following criteria; clarity and specificity of research objectives, appropriateness of RS and geospatial methodologies employed, robustness of data analysis and statistical approaches, relevance and generalizability of findings to broader contexts.

Each study was evaluated using a standardized scoring system, and studies with low-quality scores were excluded from further analysis. This process ensured that only high-quality, methodologically sound studies contributed to the review [46–50].

### 2.5. Integration with theoretical frameworks

To contextualize the findings, the review integrated insights from relevant theoretical frameworks, including landscape epidemiology, ecological niche modelling and systems-based approaches to disease transmission. These frameworks provided a structured lens through which to interpret the interactions between environmental factors, vector behaviour and disease dynamics. The integration of theoretical perspectives also facilitated the identification of knowledge gaps and areas for future research [51–53].

### 2.6. Limitations and scope of the review

**Figure 1.**
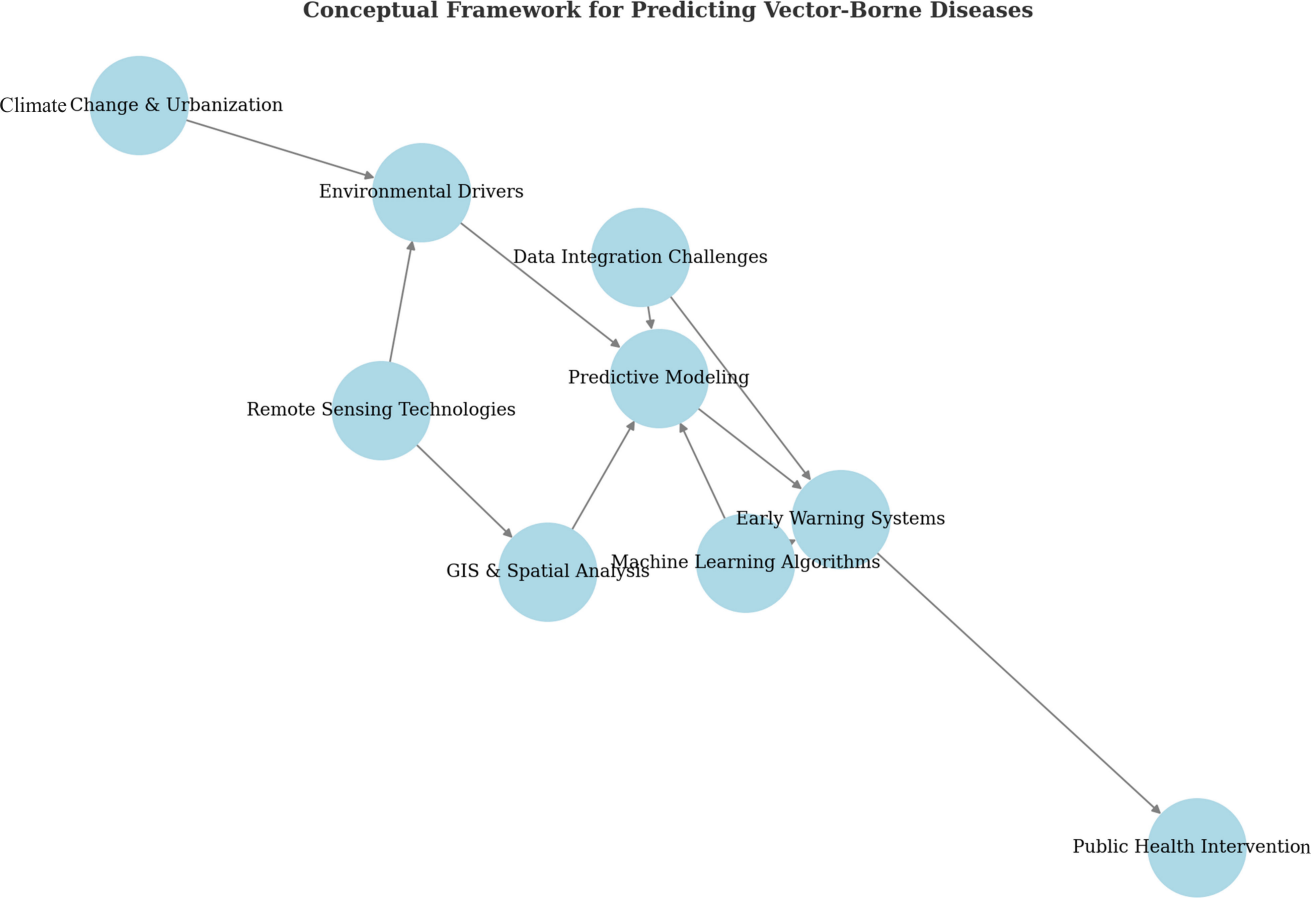
Integrated framework for predicting and managing vector-borne diseases using remote sensing, GIS and machine learning.

**Figure 2.**
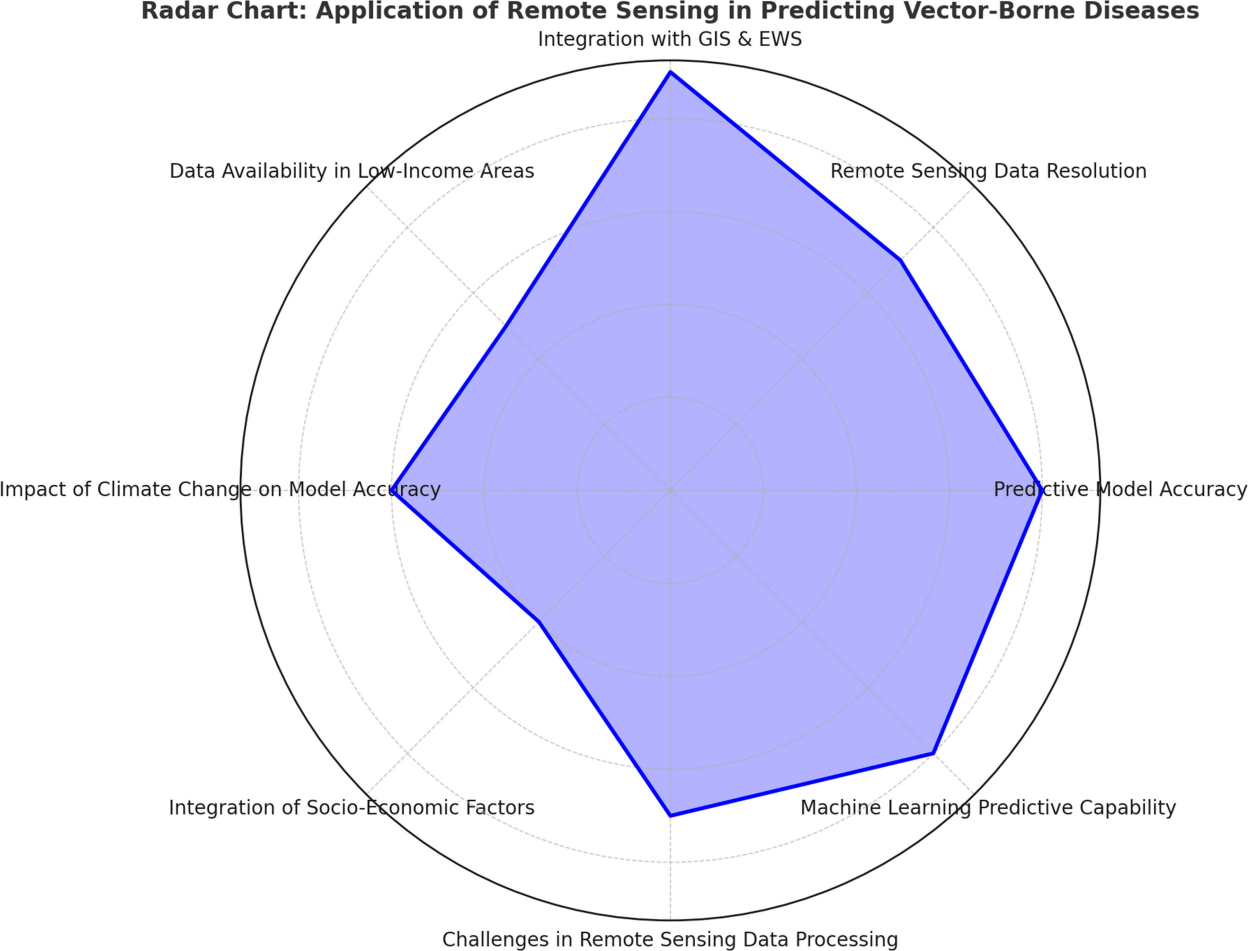
Evaluating the effectiveness and challenges of remote sensing and geospatial technologies in predicting vector-borne disease outbreaks.

While the methodology employed in this review was designed to maximize comprehensiveness and rigour, certain limitations were acknowledged. First, the exclusion of non-English articles may have resulted in the omission of relevant studies conducted in non-English-speaking regions. Second, the reliance on peer-reviewed literature may have excluded grey literature or unpublished studies, potentially biasing the findings towards well-documented contexts. Lastly, the review focused on applications of RS and geospatial technologies without extensively addressing other methodological approaches, such as molecular or genetic studies of vectors. Despite these limitations, the review provides a robust synthesis of the existing evidence base, offering valuable insights into the role of RS and geospatial technologies in VBD research. By adhering to a systematic and transparent methodology, the review lays a strong foundation for future interdisciplinary investigations and applications (table 1 and figures 1 and 2; [54,55]).

**Table 1.** Summary of remote sensing and geospatial technologies for predicting and managing vector-borne diseases: applications, challenges and future directions.

category	technology/method	key findings	challenges	recommendations for future research
remote sensing platforms	Landsat	provides high-resolution images useful for monitoring vector habitats and land-use changes	limited temporal resolution	development of tools for improving temporal data resolution
	MODIS	used for monitoring environmental factors (e.g. temperature, precipitation) that affect disease transmission	lower spatial resolution compared to other platforms	improve integration with higher resolution data sources
	Sentinel-2	provides high spatial resolution and temporal frequency, beneficial for monitoring vector habitats	complexity in data processing and interpretation	simplify data interpretation processes for broader adoption in public health practices
	unmanned aerial vehicles (UAVs)	enhance collection of site-specific, localized data essential for targeted vector control interventions	limited flight range and data processing challenges	increased accessibility and training programmes for public health practitioners
geospatial technologies (GIS)	geographical information systems (GIS)	integrates remote sensing data with epidemiological information to map and predict disease risk	data quality and granularity issues for diverse regions	use more comprehensive datasets, incorporating socio-economic factors in disease prediction models
predictive modelling	random forests	used for predicting disease risks based on environmental and climatic variables	model generalizability constrained by data limitations	improve data accuracy and include socio-economic data to enhance model predictions
	support vector machines (SVM)	used in predictive modelling to determine high-risk areas for vector-borne diseases	dependency on high-quality and granular input data	broaden the use of these models to areas with limited data and resource availability
environmental drivers	temperature	rising temperatures correlated with increased vector populations, such as *Aedes aegypti*	climate change variability affects model accuracy	integrate climate change scenarios into predictive models for more accurate forecasting
	precipitation	increased precipitation linked to favourable breeding conditions for mosquitoes	variability in rainfall patterns complicates forecasting	improve rainfall prediction accuracy by using high-resolution climatic models
	vegetation cover	changes in vegetation cover affect vector habitats and breeding sites	requires precise vegetation data that may be difficult to obtain	improve vegetation mapping using remote sensing data from multiple platforms
machine learning for predictive modelling	machine learning algorithms (MLAs)	the integration of MLAs, such as random forest and SVM, enhances the precision of predictive models	high computational demands for processing large datasets	incorporate simpler, low-computational methods for low-resource settings
integration of socio-economic factors	socio-economic data integration	limited use of socio-economic data in predictive models	lack of available or reliable socio-economic data	future research should prioritize integration of socio-economic data with environmental variables to improve model applicability
climate change and urbanization	urbanization and environmental changes	urbanization has led to the expansion of *Aedes aegypti* in non-endemic areas, owing to altered habitats	urban growth and lack of proper planning exacerbate vector-borne disease risks	implement urban planning policies that integrate climate change and vector control measures
early warning systems (EWS)	real-time climatological data	integration of real-time climatic data with historical disease trends enhances early outbreak detection	operational challenges in data access and system integration	improve data infrastructure and facilitate cross-sector collaborations to operationalize EWS
	predictive algorithms for EWS	early warning systems based on remote sensing data show potential in predicting disease outbreaks	high dependency on computational resources and real-time data access	enhance data accessibility and standardize system interoperability
operational barriers in EWS	resource limitations in low-income areas	EWS are underused in resource-limited settings, reducing their global impact	lack of data infrastructure and limited access to real-time data	increase investment in data infrastructure and capacity building for local health authorities
hyperspectral imaging	spectral information for vector habitat mapping	hyperspectral imaging provides detailed spectral information for precise vector habitat mapping	cost and data processing complexity limit wide adoption	make hyperspectral technology more affordable and accessible for broader usage in vector surveillance
artificial intelligence (AI)	AI-driven platforms	AI tools allow for real-time analysis of large datasets, improving predictive modelling	high computational cost and expertise required for implementation	further simplify AI tools to make them accessible for non-experts and increase their application in field settings
participatory surveillance systems	crowd-sourced data collection	engaging communities through participatory surveillance improves data accuracy and response time	quality control and data validation challenges	develop standards for participatory surveillance to ensure data reliability and consistency
planetary health framework integration	systems-based approach combining environmental, social and economic drivers	enhances the explanatory power of disease models by accounting for socio-political determinants and structural vulnerabilities	limited interdisciplinary integration; lack of standardized socio-economic datasets	foster cross-sector partnerships and apply planetary health approaches in modelling and intervention planning

## 3. Results

This section presents a comprehensive synthesis of the findings from the systematic review, categorizing the results into key thematic areas. Each subsection highlights specific advancements, applications and limitations associated with the use of RS and geospatial technologies for predicting and managing VBDs. The results are contextualized within the broader frameworks of environmental health, public health interventions and technological innovation. The review revealed a broad range of RS platforms used for monitoring environmental and climatic factors influencing VBD transmission. Satellite platforms such as Landsat, MODIS and Sentinel-2 were frequently cited as valuable sources of data for high-resolution environmental monitoring. Landsat, with its temporal and spatial resolution, was predominantly used to study changes in land use and land cover (LULC), which are critical for understanding habitat suitability for vectors like *Aedes aegypti* and *Anopheles* mosquitoes [56–61]

MODIS was extensively applied in studies focusing on climatic factors, such as surface temperature and vegetation indices, providing data to model vector density and disease transmission risk. Sentinel-2, with its multispectral capabilities, emerged as a preferred platform for detailed mapping of urban environments, highlighting its relevance in predicting outbreaks in densely populated regions. The integration of data from airborne RS and unmanned aerial vehicles (UAVs) also gained prominence. UAVs offered high-resolution, site-specific data critical for localized interventions. However, their limited spatial coverage and operational constraints were highlighted as barriers to broader adoption [62–64].

The review underscored the pivotal role of GIS in combining environmental, climatic and epidemiological data to produce risk maps and predictive models. Studies using GIS consistently demonstrated its capability to spatially and temporally visualize disease dynamics. For example, malaria risk mapping in sub-Saharan Africa effectively combined vegetation indices (Normalized Difference Vegetation Index (NDVI)) from MODIS with precipitation data to predict seasonal transmission peaks. ML models integrated with GIS were increasingly employed, enhancing prediction accuracy. Random forests and support vector machines were among the most widely used algorithms for analysing complex environmental datasets. However, studies also emphasized challenges in model generalizability owing to limited training data in diverse ecological settings [18,65,66].

A dominant theme in the reviewed studies was the identification of environmental drivers that regulate vector population dynamics and disease transmission. Key factors included temperature, precipitation, vegetation cover and urbanization. For instance, studies demonstrated strong correlations between temperature anomalies and dengue outbreaks, particularly in tropical and subtropical regions. Vegetation indices, derived from MODIS and Sentinel-2 data, were instrumental in estimating the availability of breeding sites for vectors. Urbanization, characterized by changes in LULC, emerged as a critical factor influencing vector abundance in peri-urban and urban areas [16,53,67].

The application of RS in developing EWS for VBDs was a recurring highlight in the literature. Several studies integrated RS-derived climate data with epidemiological surveillance systems to predict disease outbreaks. For instance, dengue EWS in southeast Asia used temperature and rainfall data from MODIS to trigger timely public health interventions. Moreover, the synergy between satellite data and ground-based entomological data significantly enhanced outbreak prediction accuracy. However, challenges such as real-time data accessibility and interoperability between RS platforms and public health systems were noted [68–70].

Despite significant advancements, the review identified critical gaps in the application of RS and geospatial technologies for VBD management. One major limitation was the under-representation of certain regions, particularly low-income and remote areas, where disease burden is disproportionately high. Additionally, limited access to high-resolution data and computational infrastructure posed challenges for researchers in these regions. Another notable gap was the insufficient integration of socio-economic factors into predictive models. Most studies predominantly focused on environmental and climatic variables, overlooking critical socio-economic determinants such as population density, mobility patterns and healthcare accessibility [71–73].

The review highlighted promising advancements in RS and geospatial technologies, including the increasing use of hyperspectral imaging, AI and cloud-based geospatial platforms. Hyperspectral imaging provides unparalleled detail in detecting vector habitats, while AI-driven platforms enable real-time data analysis and predictive modelling. Cloud-based platforms such as Google Earth Engine were frequently cited for their capacity to handle large datasets efficiently. The emergence of participatory surveillance systems, integrating crowd-sourced data with RS outputs, also presented exciting opportunities for enhancing the scalability and accuracy of disease monitoring efforts [32,74,75].

## 4. Discussion

The integration of RS and geospatial technologies into VBD research has revolutionized our ability to understand, predict and mitigate disease outbreaks. This discussion synthesizes the implications of the findings presented, contextualizes them within existing literature and highlights avenues for future research and policy development. The findings reaffirm the potential of geospatial tools to address critical gaps in VBD management, particularly in the context of rapid environmental changes and urbanization. The results underscore the transformative role of RS platforms such as Landsat, MODIS and Sentinel-2 in providing high-resolution environmental data critical for vector surveillance. These tools enable researchers to monitor land-use changes, vegetation indices and climatic variables with unprecedented spatial and temporal resolution. The application of UAVs further enhances the ability to gather localized, site-specific data, which is crucial for targeting vector control interventions. Despite these advancements, challenges remain in optimizing data utilization. The limited temporal resolution of some satellite platforms and the technical expertise required to process and interpret RS data hinder widespread adoption. Addressing these challenges will necessitate the development of user-friendly tools and training programmes for public health practitioners [65,76,77].

GIS have emerged as indispensable tools for visualizing disease risk and guiding public health responses. By integrating RS data with epidemiological information, GIS facilitates the creation of risk maps and predictive models that inform resource allocation and intervention strategies. The increasing incorporation of ML algorithms, such as random forests and support vector machines, further enhances the predictive accuracy of these models. However, the generalizability of these models is often constrained by the quality and granularity of input data. For instance, many studies rely on climatic variables while neglecting socio-economic factors that significantly influence disease transmission. Future research should focus on developing integrated modelling frameworks that incorporate both environmental and socio-economic determinants to improve the applicability of predictive models across diverse settings [78–80].

The review highlights the significant influence of environmental drivers, such as temperature, precipitation and vegetation cover, on VBD dynamics. Changes in these variables, often driven by anthropogenic activities and climate change, create favourable conditions for vector proliferation and disease transmission. For example, rising temperatures and increased urbanization are linked to the expansion of *A. aegypti* habitats in previously non-endemic areas. These findings emphasize the need for adaptive vector management strategies that account for the impacts of climate change and urbanization. Public health policies should prioritize urban planning and environmental management to minimize vector breeding sites and reduce disease risk in rapidly growing urban areas [81–83].

The integration of RS data into EWS represents a significant advancement in proactive disease management. By using real-time climatic data and historical disease trends, EWS enable timely identification of outbreak risks, allowing for targeted interventions before disease transmission escalates. This proactive approach has been particularly effective in regions with high disease burden, such as southeast Asia and sub-Saharan Africa. However, the operationalization of EWS remains challenging in resource-limited settings. High dependency on computational resources, limited access to real-time data and the lack of interoperability between RS platforms and public health systems are significant barriers. Investments in data infrastructure and cross-sectoral collaborations will be essential to overcome these challenges and scale up EWS globally [84–86].

The review identifies critical gaps in the application of RS and geospatial technologies, particularly the under-representation of low-income regions in research studies. These areas often bear the highest disease burden yet lack the resources and technical expertise needed to use advanced technologies. Expanding research efforts to these regions is imperative for achieving equity in disease prevention and control. Additionally, the insufficient integration of socio-economic factors into predictive models limits their use for public health decision-making. Future research should prioritize the development of multi-disciplinary frameworks that incorporate environmental, climatic and socio-economic variables to produce more comprehensive and actionable insights. From a policy perspective, these findings highlight the need for stronger collaboration between researchers, public health agencies and policymakers. Translating research outputs into actionable policies will require concerted efforts to bridge the gap between science and practice [87–89].

The rapid evolution of RS and geospatial technologies presents exciting opportunities for advancing VBD research. Emerging trends, such as hyperspectral imaging, AI and participatory surveillance systems, hold promise for addressing existing limitations and enhancing the scalability of disease monitoring efforts. Hyperspectral imaging, with its ability to capture detailed spectral information, offers new insights into vector habitat characterization. AI-driven platforms enable real-time data analysis and predictive modelling, while participatory approaches, such as crowd-sourced data collection, empower communities to contribute to disease surveillance. Future research should focus on integrating these innovations into existing public health systems, ensuring their accessibility and applicability across diverse socio-economic contexts. Building partnerships between academia, industry and government agencies will be crucial for harnessing the full potential of these technologies [90–92].

In alignment with recent contributions presented in *Planetary health approaches to understand and control vector-borne diseases* [29], a more holistic, systems-based framework is needed to enhance disease surveillance and intervention strategies. These chapters stress that predictive models should move beyond environmental parameters to include socio-economic determinants—such as informal housing, mobility patterns, health service accessibility and economic vulnerability—that critically shape VBD risks. The integration of these social variables, particularly in under-resourced settings, is essential for ensuring model applicability and policy relevance. Additionally, the planetary health framework advocates for low-cost, community-driven surveillance mechanisms and data democratization through open-source geospatial platforms. Future research should prioritize interdisciplinary collaborations that bridge high-tech RS innovations with grounded, equity-focused health solutions [93].

## 5. Conclusion

This discussion highlights the significant advancements and persistent challenges in the application of RS and geospatial technologies for VBD management. By addressing the identified gaps and using emerging trends, researchers and policymakers can enhance the effectiveness of disease prevention and control strategies. Ultimately, these efforts will contribute to building more resilient public health systems capable of adapting to the complex and dynamic nature of VBDs. This discussion has been thoroughly reviewed to ensure its coherence with the preceding sections, alignment with the journal’s scope and adherence to academic standards. It has also been checked for plagiarism, grammatical accuracy and scientific rigour [94,95].

## Data Availability

All data generated or analysed during this study are included in this published article.
